# Flow Cytometry Analysis of Blood Large Extracellular Vesicles in Patients with Multiple Sclerosis Experiencing Relapse of the Disease

**DOI:** 10.3390/jcm11102832

**Published:** 2022-05-17

**Authors:** Jakub Soukup, Marie Kostelanská, Sami Kereïche, Andrea Hujacová, Miluše Pavelcová, Jiří Petrák, Eva Kubala Havrdová, Karel Holada

**Affiliations:** 1 Institute of Immunology and Microbiology, First Faculty of Medicine, Charles University, 128 00 Prague, Czech Republic; jakub.soukup@lf1.cuni.cz (J.S.); marie.kostelanska@lf1.cuni.cz (M.K.); ajka.hujacova@gmail.com (A.H.); 2 Department of Genetics and Microbiology, Faculty of Science, Charles University, 128 44 Prague, Czech Republic; 3 Institute of Biology and Medical Genetics, First Faculty of Medicine, Charles University, 128 00 Prague, Czech Republic; sami.kereiche@lf1.cuni.cz; 4 Department of Neurology and Clinical Neuroscience, First Faculty of Medicine, Charles University and General University Hospital, 128 21 Prague, Czech Republic; miluse.pavelcova@vfn.cz (M.P.); eva.havrdova@gmail.com (E.K.H.); 5 BIOCEV, First Faculty of Medicine, Charles University, 252 50 Vestec, Czech Republic; jiri.petrak@lf1.cuni.cz

**Keywords:** extracellular vesicles, plasma, multiple sclerosis, flow cytometry, cryo-electron microscopy

## Abstract

The number of people living with multiple sclerosis (MS) in developed countries is increasing. The management of patients is hindered by the absence of reliable laboratory tests accurately reflecting the disease activity. Extracellular vesicles (EVs) of different cell origin were reportedly elevated in MS patients. We assessed the diagnostic potential, with flow cytometry analysis, of fresh large EVs (lEVs), which scattered more light than the 590 nm silica beads and were isolated from the blood plasma of relapsing remitting MS patients. Venous blood was collected from 15 patients and 16 healthy controls (HC). The lEVs were isolated from fresh platelet-free plasma by centrifugation, labelled with antibodies and the presence of platelet (CD41+, CD36+), endothelial (CD105+), erythrocyte (CD235a+), leukocyte (CD45+, CD19+, CD3+) and phosphatidylserine (Annexin V+) positive lEVs was analyzed using standard flow cytometry. Cryo-electron microscopy was used to verify the presence of EVs in the analyzed plasma fractions. MS patients experiencing acute relapse had slightly reduced relative levels (% of positive lEVs) of CD105+, CD45+, CD3+, CD45+CD3+ or CD19+ labelled lEVs in comparison to healthy controls. An analysis of other markers or a comparison of absolute lEV counts (count of lEVs/µL) did not yield any significant differences. Our data do not support the hypothesis that the exacerbation of the disease in RRMS patients leads to an increased numbers of circulating plasma lEVs which can be monitored by standard flow cytometry.

## 1. Introduction

Multiple Sclerosis (MS) is the most common and incurable cause of neurologic disability in young adults in Western European countries. It is an autoimmune disease characterized by the demyelination of neurones in the central nervous system which seems to correlate with the migration of inflammatory cells through the blood–brain barrier (BBB) [1]. Recently, a number of new disease-modifying drugs have been introduced; mostly targeting cells of the immune system, but their effective use in MS management is hindered by the absence of reliable, sensitive and specific laboratory tests accurately reflecting the disease activity [2]. The disease has three main forms: relapsing and remitting MS (RRMS, ~90% of cases) in which periods of neurological deterioration are interspersed with periods of stability; primary progressive MS (PPMS, ~10% of cases) in which neurological decline progresses steadily from the disease onset; and secondary progressive MS (SPMS) which develops in many relapsing and remitting patients within 10 years of the disease manifestation. RRMS patients are mostly treated with anti-inflammatory therapies such as interferon-beta, fingolimod or Natalizumab [3,4]. Our study aimed to evaluate the diagnostic potential of the standard flow cytometry of freshly isolated large blood extracellular vesicles to confirm acute relapse of the disease in RRMS patients.

Extracellular vesicles are heterogeneous in their size, composition and biogenesis. The main types of EVs are represented by exosomes, microvesicles and apoptotic bodies. Exosomes are vesicles with sizes of 30–150 nm, which is too small for an analysis by standard flow cytometry. Microvesicles (MVs; e.g., ectosomes, shedding vesicles) are lipid particles which are shed from the plasma membrane and their size, from 100 to 1000 nm, is partially within the sensitivity of standard flow cytometry [5,6,7]. MVs are formed by the outward budding of the plasma membrane assisted by cytoskeleton changes. MVs tend to be enriched in specific membrane proteins [8] which provide instrument for the recognition of their cellular origin in body liquids [7,9,10]. In recent years, EVs have been studied in correlation with a number of diseases [11,12,13] and their possible involvement in neurodegenerative disorders was recently reviewed [14]. The role of EVs in the pathogenesis of MS remains controversial, and both protective and detrimental effects have been proposed [7]. A number of previous studies utilized flow cytometry to evaluate the presence EVs in blood, cerebrospinal fluid and recently in the tears of MS patients [15,16]. Several studies on blood plasma reported elevated numbers of endothelial, platelet or leukocyte EVs. Minagar et al. reported an elevation of endothelial EVs labelled with PECAM-1 (CD31) antibody in the plasma of MS patients in relapse, but not in remission. Integrin αV (CD51)-labelled EVs were elevated both in relapse and in remission [17]. Jy et al. observed a high elevation of endothelial E-selectin (CD62E)-positive EVs, but not in remission. However, the number of ICAM-1 (CD54)-positive EVs did not differ from healthy controls. Treatment of RRMS patients with interferon β leads to a gradual decrease in the number of plasma endothelial CD31+ EVs over time [18,19]. Marcos-Ramiro et al. reported the elevation of plasma CD31+ EVs, but not CD62E+ EVs, in all clinical forms of MS. In addition, they also observed a universal elevation of platelet EVs labelled with platelet GPIbα (CD42b) antibody [20]. Higher levels of platelet CD41 (integrin αIIb) or CD61 (integrin β3)-positive EVs in untreated MS patients were also reported [21,22]. In addition, higher levels of plasma CD61+ EVs were found in RRMS compared to SPMS patients. Interestingly, the levels of leukocyte CD45+ (Protein Tyrosine Phosphatase Receptor Type C) and monocyte CD14+ (monocyte differentiation antigen CD14)-labelled EVs increased both in RRMS and SPMS, but not in untreated MS patients. RRMS patients treated with Natalizumab or interferon β had higher plasma levels of all the studied EVs (CD61+, CD14+, CD45+) compared to untreated patients [22]. Triple labelling of plasma endothelial EVs with CD31, CD51/61 (integrin αV/β3) and CD54 demonstrated increased levels of activated endothelial EVs (CD31+CD51/61+CD54+) in RRMS, but not in SPMS patients. In contrast, the number of CD31+CD51/61-CD54- EVs did not change or were lower in RRMS and SPMS patients, respectively [23].

The aim of our study was to evaluate whether the acute exacerbation of symptoms in RRMS patients is connected with changes in the usual number of large cell-specific EVs circulating in the blood. We analyzed the level of platelet (CD41+, CD36+), endothelial (CD105+), erythrocyte (CD235a+), leukocyte (CD45+), B-lymphocyte (CD19+) and T-lymphocyte (CD3+) EVs. From the technical standpoint, we analyzed fresh large EVs (lEVs) pelleted at 14,000 g and with a size of above the sensitivity limit of standard flow cytometry.

## 2. Materials and Methods

### 2.1. Patients

All patients (MS) and healthy controls (HC) signed an informed consent form which was approved by Ethics committee of the General University Hospital in Prague (approval no. 120/14). A total of 15 patients diagnosed with RRMS (10 females, 5 males, mean age 39.7 ± 8.9 years) were included in this study alongside 16 healthy blood donors (7 females, 9 males, mean age 45.5 ± 10.5 years). All HCs were recommended a low-fat diet prior to blood donation. MS patients were not provided with any dietary recommendations before blood collection. All patients underwent medical examination to confirm the recent exacerbation of the disease which started on average 7.5 ± 4.5 days before blood collection and was defined as worsening in terms of current symptoms or the appearance of new symptoms lasting at least 24 h without concurrent infection or overheating of the organism. The mean value of the Kurtzke Expanded Disability Status Scale used to assess the severity of the symptoms was 2.8 ± 0.6. All patients, except 2, were treated with disease-modulating drugs: 7 with fingolimod, 2 with glatiramer acetate, 2 with interferon beta-1a, 1 with interferon beta-1b and 1 with natalizumab. Four patients had intravenous corticosteroids 4–13 days before the blood collection. Venous blood samples were collected in a BD Vacutainer tube with K2EDTA and analyzed immediately. The analysis of the patient sample was accompanied by a parallel analysis of a sample of the healthy donors.

### 2.2. Antibodies and Solutions

The following antibodies and solutions were obtained from BD Biosciences (NJ, USA): Lysing Solution 10× Concentrate (cat. no. 349202); Mouse monoclonal antibody (mAb) CD105 PE (IgG1, clone 266)—used for lEVs analysis; mAb CD4 PE (IgG1, clone SK3). Annexin V Binding Buffer was obtained from Abcam (cat. no. ab14085, Cambridge, UK). The following were obtained from Exbio (Vestec, Czech Republic): Mouse IgG1 Isotype Control FITC (clone MOPC-21); Mouse IgG1 Isotype Control PE (clone PPV-06); mAb CD3 FITC (IgG2a, clone MEM-57); mAb CD19 APC (IgG1, clone LT19); mAb CD36 FITC (IgG1 clone TR9); mAb CD41 PE (IgG1, clone MEM-06); mAb CD45 Pacific Blue (IgG1, clone MEM-28); mAb CD235a PE (IgG2b, clone HIR-2) and mAb CD105 PE (IgG2a, clone MEM-226)—used for the lEV-deprived plasma analysis; Annexin V-FITC (cat. no. EXB0024). The ApogeeMix bead mixture (cat. No. 1493) was obtained from Apogee Flow Systems (Northwood, UK). All utilized mAbs, with the exception of CD105 PE, were titrated to estimate their saturating concentration using anticoagulated blood of a healthy donor. CD105 PE mAbs were used undiluted.

### 2.3. Isolation of Fresh lEVs

**Figure 1:**
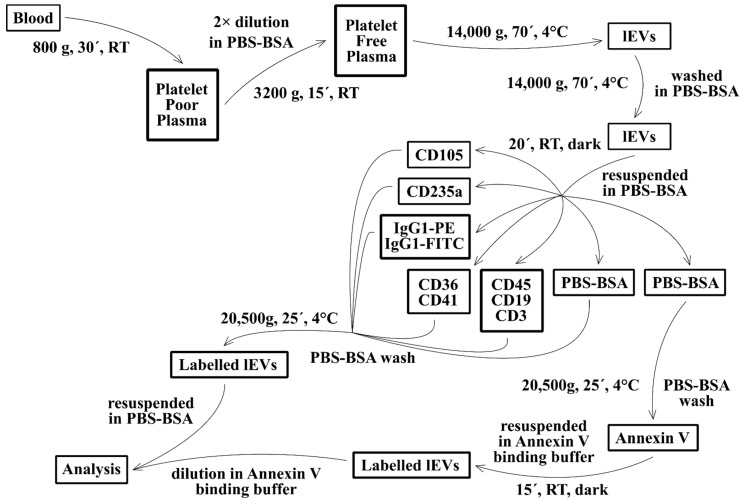
Scheme of isolation and labelling of fresh large extracellular vesicles (lEVs) from blood. Each time one patient and one healthy control blood sample were processed simultaneously. RT—room temperature; PBS-BSA—phosphate buffered saline pH 7.4 with 0.1% bovine serum albumin.

The isolation of lEVs from 4.5 mL of K_2_EDTA anticoagulated blood of the MS patients and HCs started within 20 min from collection. Blood was transferred into a 13 mL polypropylene Sarstedt tube and centrifuged at 800× *g*, 22 °C for 30 min in the Eppendorf 5810R centrifuge (swing rotor A-4-62, Hamburg, Germany). A total of 2 mL of platelet-poor plasma was transferred into a new tube and diluted with an equal volume of 100 nm filtered PBS pH 7.4 with 0.1% BSA (PBS-BSA) to lower the viscosity of the sample [24]. Diluted plasma was centrifuged at 3200× *g*, 22 °C for 15 min to pellet the remaining platelets. Platelet-free plasma was centrifuged at 14,000× *g* and at 4 °C for 70 min (fixed rotor F34-6-38), and supernatant was frozen on dry ice for further analysis. Pelleted lEVs were resuspended in 0.5 mL of PBS-BSA, transferred to a 1.5 mL Eppendorf tube and sedimented by centrifugation at 14,000× *g*, 4 °C for 70 min (rotor F45-30-11). Washed, fresh lEVs were resuspended in 350 µL of PBS-BSA and the aliquots were immediately utilized for labelling (Figure 1).

### 2.4. lEVs Labelling

**Figure 2:**
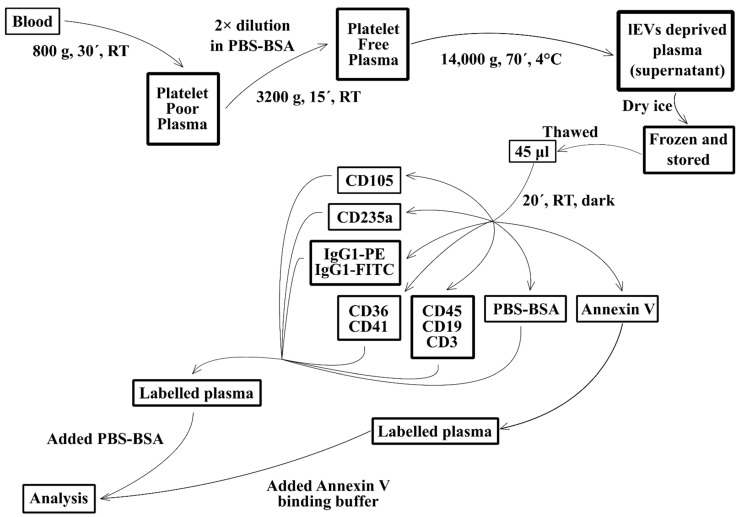
Scheme of preparation and labelling of plasma deprived of lEVs by centrifugation. Each time one patient and one healthy control plasma sample were processed simultaneously. RT—room temperature; PBS-BSA—phosphate buffered saline pH 7.4 with 0.1% bovine serum albumin.

Next, 45 µL of lEV suspension was labelled with 15 µL (5 µL of each mAb was supplemented with buffer to a final volume of 15 µL) of mAb solutions containing: CD105 PE (undiluted); CD235a PE (16 μg/mL); CD36 FITC (0.22 μg/mL) + CD41 PE (5 μg/mL); CD3 FITC (2.5 μg/mL)+ CD19 APC (2.92 μg/mL) + CD45 Pacific Blue (8.75 μg/mL); IgG1 FITC (0.22 μg/mL) + IgG1 PE (5 μg/mL); or PBS-BSA. Samples were incubated in the dark at RT for 20 min. All samples were washed with 1 mL of PBS-BSA and centrifuged at 20,500× *g*, and 4 °C for 25 min (rotor F45-30-11). Mab-labelled samples were resuspended in 200 µL PBS-BSA and one non-labelled aliquot was resuspended in 50 µL of 100 nm filtered Annexin-V binding buffer (ABB) with Annexin-V-FITC, incubated in the dark at RT for 20 min and diluted with 150 µL ABB (Figure 1). The analysis with flow cytometry was carried out immediately. The labelling of frozen plasma supernatants depleted of lEVs is described in Figure 2.

### 2.5. Flow Cytometry Analysis

The BD FACSCantoTM II cytometer was set to a low flow rate (~10 µL/min) and the threshold was lowered to minimum (FSC and SSC threshold 200 with parameter “AND”) with the voltage set to 600 for FSC and 500 for SSC. ApogeeMix beads were used to construct the lEVs gate within the sensitivity limit of the cytometer and buffers were used for dilution. Samples were checked for background noise level. Samples of one patient and one control were analyzed simultaneously each day. The actual flow rate (9.2 µL/min) was estimated using manually counted Fluoresbrite calibration beads after the completion of the study (Text S1). Each sample was collected for 2 min (equivalent of 18.4 µL). The preparation and analysis of fluorescence-compensation samples and of frozen plasma supernatants depleted of lEVs is described in Text S2 and Text S3.

### 2.6. FACS Light Scatter Calibration

The ApogeeMix beads data were used to calibrate light scatter using freely available FCMPass software v3.07 as published by Welsh et al. [25]. The preference for high refractive index (RI) EVs, predefined in the software, was used for the calculations. The bead catalogue was created based on the ApogeeMix beads datasheet using the RI of the beads. The 1300 nm, 880 nm and 590 nm silica beads (RI = 1.43) and 500 nm latex beads (RI = 1.59) were used for the calibration.

### 2.7. Transmission Cryo-Electron Microscopy (Cryo-TEM)

Cryo-TEM measurements were carried out on a Tecnai G2Sphera 20 electron microscope (Thermo Fisher, Waltham, MA, USA) equipped with a Gatan 626 cryo-specimen holder and an LaB6 gun. The samples were prepared by plunge-freezing [26]. Briefly, 3 μL of the sample, either isolated lEVs or plasma supernatant depleted of lEVs, was applied to a copper grid covered with a perforated carbon film forming woven-mesh-like openings of different sizes and shapes (the lacey carbon grids #LC-200 Cu, Electron Microscopy Sciences, Hatfield, PA, USA), which was then glow discharged for 40 s with a 5 mA current prior to specimen application. Most of the sample was removed by blotting (Whatman no. 1 filter paper) for approximately 1 s, and the grid was immediately plunged into liquid ethane held at −183 °C. The grid was transferred without rewarming into the microscope. Images were recorded at an accelerating voltage of 120 kV and with magnifications ranging from 6000× to 29,000× using a GatanUltraScan 1000 slow-scan CCD camera. All cryo-TEM pictures were carefully inspected for possible artefacts such as radiation damage and ice crystals, and high-quality images were CTF-corrected and band-pass filtered in order to suppress both ice thickness variations and noise to below a 1 nm detail size.

### 2.8. Data Evaluation and Statistics

**Figure 3:**
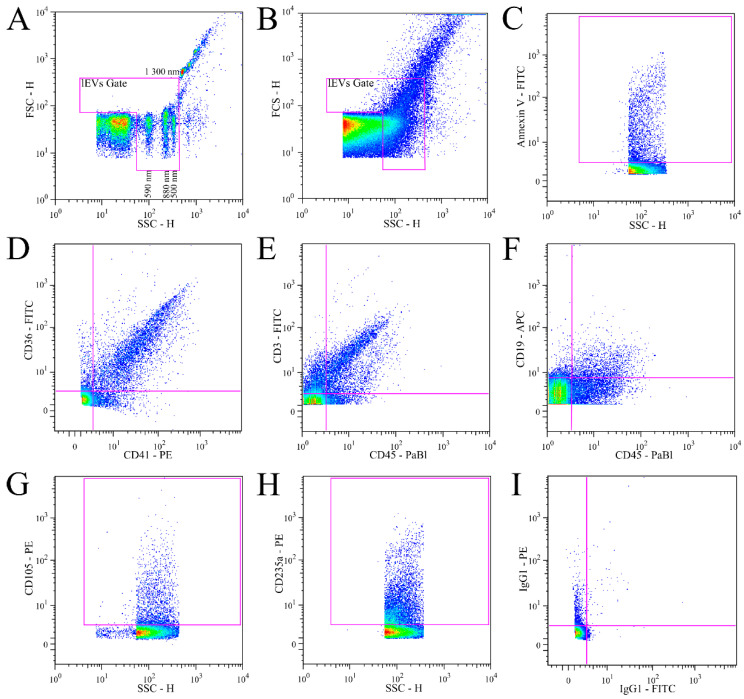
Definition of lEVs gate and representative results of lEVs labelling. (**A**). ApogeeMix beads were used to set lEVs gate to size of the beads. The mix consisted of 180 nm, 240 nm, 300 nm (below sensitivity), 590 nm, 880 nm and 1,300 nm silica beads with refractive index 1.43 and 2 latex beads (110 nm and 500 nm) with green fluorescence and refractive index 1.59. (**B**). An illustrative scattergram of isolated plasma vesicles with lEVs gate. Only events in the lEVs gate were included in the analysis. (**C**–**I**). Illustrative fluorescence dot plots showing representative results of lEVs labelling with: Annexin V FITC (**C**), CD36 FITC + CD41 PE (**D**), CD45 PaBl + CD3 FITC (**E**), CD45 PaBl + CD19 APC (**F**), CD105 PE (**G**), CD235a PE (**H**) and isotypic control IgG1 FITC + IgG1 PE (**I**).

Measured data were analyzed in FlowJo ver. 8.8.6. (BD Biosciences, Ashland, OR, USA). The gate for analysis of lEVs was set up using ApogeeMix Beads (Figure 3). The silica beads with a size of 590, 880 and 1300 nm and 500 nm fluorescent polystyrene beads formed separate populations on the scattergram, but smaller silica beads of 180, 240 and 300 nm in size were not distinguished, and their signal marked the lower limit of the lEVs gate. The upper limit of the lEVs gate was set up exclude 1300 nm silica beads. The threshold of fluorescence-positive events was set on an isotypic IgG negative control for washed lEVs samples. The number of measured events was calculated as number of events per µL using the measured flow rate (9.2 µL/min) and known time of acquisition. Compensations were performed using the FlowJo compensation wizard.

A statistical analysis was performed in GraphPad Prism 5 version 5.03 for Windows (GraphPad Software, San Diego, CA, USA). To compare MS samples with HC samples, we used unpaired two-tailed Mann–Whitney test. A nonparametric test was used since some of the data failed to pass D’Agostino and Pearson’s omnibus normality test. The significance level was set to *p*-value < 0.05 (* *p* < 0.05; ** *p* < 0.005). Data are stated as the mean value with a 95% confidence interval.

## 3. Results

### 3.1. Validation of lEVs Flow Cytometry Analysis

To assess the linearity of the flow cytometry measurement, we analyzed binary dilutions of lEVs isolated from three individual donors and labelled them with CD36 and CD41 (Figure S1). A decrease in the event counts (full lines) corresponded to the binary dilutions of samples demonstrating the ability of the assay to detect changes in the count of labelled lEVs. The median of fluorescence intensity of the detected events decreased slightly with dilutions in two samples. The third sample demonstrated a more pronounced decrease in fluorescence, suggesting the possible presence of coincident “swarm” detection [27]. To validate our analytical procedure, we analyzed three separate samples, each in three technical replicates of isolation and labelling. Isolated lEVs were labelled with mAbs against platelet markers CD36 and CD41 or with Annexin V. The differences among technical replicates were within 7% of the mean value in experiments 1 and 3, but the difference was higher, at up to 29% for Annexin V and up to 19% for double CD36 and CD41-labelled replicates in experiment 2 (Figure S1B–D). To determine the size of the detected vesicles, we retrospectively calibrated the SSC signal using ApogeeMix beads. The calibrated size of lEVs in the gate ranged from approximately 650 nm to 2000 nm. A comparison of the arbitrary units (SSC-H) of our cytometer and standard units (nm) is shown in Figure S2.

### 3.2. Transmission Cryo-Electron Microscopy of Isolated lEVs

**Figure 4:**
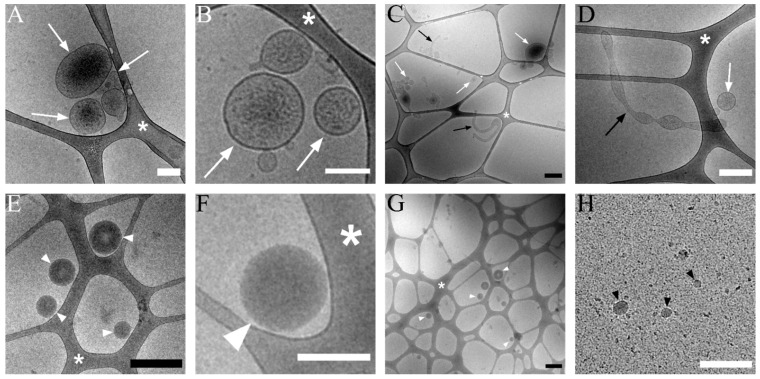
Cryo-TEM of isolated lEVs (upper row) and supernatant plasma deprived of lEVs (lower row). (**A**,**B**) A detailed picture of lEVs (white arrow). (**C**). Overview of lEVs sample. lEVs (white arrow), elongated vesicle (black arrow) (**D**). Elongated vesicles (black arrow) which occasionally present in isolated lEVs (white arrow). (**E**). Electron-dense vesicles commonly present in plasma supernatant after lEVs isolation (white arrowhead). (**F**). A detailed picture of the electron-dense vesicle (white arrowhead). (**G**). Plasma supernatant showing the electron-dense vesicles (white arrowheads) and impurities such as protein aggregates. (**H**). Small (up to 50 nm) vesicles (black arrowhead) and proteins are visible in the picture. White scale bar 200 nm, black scale bar 500 nm. Asterisk marks lacey carbon support film on the grid.

Cryo-TEM was used to visualize lEVs isolated by the procedure used in our flow cytometry analysis (Figure 1). The samples contained sedimented EVs with an approximate size from 70 nm to 500 nm (Figure 4A,B). Larger vesicles were deformed or poorly visible due to ice thickness. Occasionally, elongated vesicles with a length in the µm scale were visualized, but their width was up to 200 nm (Figure 4D). We did not detect the presence of protein or vesicle aggregates. The purity of the isolation can be seen in an overview picture presented in Figure 4C. As a control, we used the plasma supernatant from lEVs isolation. Samples of the supernatant contained electron-dense particles with a size of up to 300 nm (Figure 4E,F). The presence of impurities alongside electron-dense vesicles is apparent in images of the supernatant (Figure 4G). We also detected the presence of small vesicles (<50 nm) and proteins (Figure 4H).

### 3.3. Analysis of Isolated Fresh Plasma lEVs of MS

**Figure 5:**
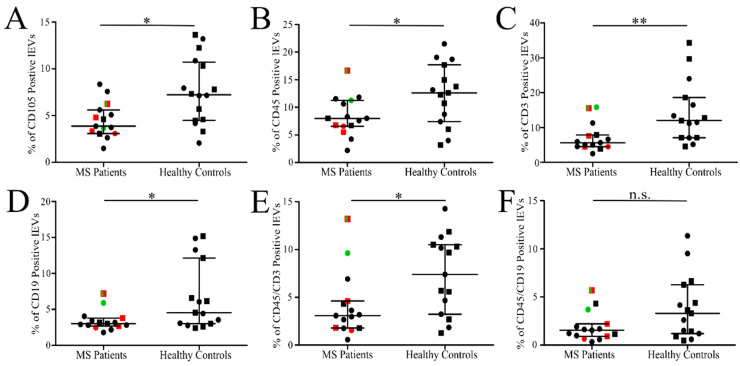
Relative number of labelled isolated lEVs in MS patients and HC. Percentage of endothelial CD105+ (**A**), leukocyte CD45+ (**B**), T-lymphocyte CD3+ (**C**) and CD45+CD3+ (**E**), B-lymphocyte CD19+ (**D**) and CD45+CD19+ (**F**) lEVs out of all events collected in lEVs gate. The line represents median value with interquartile range. Women (circles), men (squares), patients without treatment (green), patients receiving intravenous corticoids up to 14 days before blood collection (red). * *p* < 0.05, ** *p* < 0.005, n.s.—not significant; MS patients (*n* = 15) and HC (*n* = 15 or *n* = 16).

Only the events collected in the lEVs gate were included in the analysis (Figure 3). The mean number of lEVs in MS patient samples seemed higher (1620 ± 1044 particles/µL) than in HC (1116 ± 327 particles/µL) due to one outlier value (n.s.). Similarly, no significant differences in the number of fluorescence-positive events in all analyzed lEVs populations were found (Table 1 and Figure S3). In relative numbers, comparing labelled lEVs to all events in the lEVs gate, MS patients had a significantly lower number of lEVs of endothelial origin (CD105+: 4.5% vs. 7.6%; *p* = 0.0135), leukocyte origin (CD45+: 8.4% vs. 12.3%; *p* = 0.0421), B-lymphocyte origin (CD19+: 3.4% vs. 6.7%; *p* = 0.0362) and T-lymphocyte origin (CD3+: 7.1% vs. 14.3%; *p* = 0.0048 or CD45+CD3+: 4.1% vs. 7.4%; *p* = 0.0202) as shown in dot plots in Figure 5 and Figure S4. One HC sample labelled with leukocyte markers was excluded from the analysis due to measurement failure. The elevation of relative levels of lEVs labelled with platelet markers (CD36 and CD41) or with Annexin V in HC was not significant (Table 1 and Figure S4). The mean number of particles detected within the lEVs gate present in the filtered buffer used for lEVs isolation was 25 ± 11/µL and the particles did not produce a fluorescent signal above the threshold set for positive events (Figure S5).

The analysis of frozen plasma supernatants depleted of lEVs also provided no significant differences in the number of labelled events within the lEVs gate with the exception of Annexin V+ particles which were modestly higher in MS patients than in HC (241 ± 63 vs. 161 ± 39 particles/µL; Text S4, Table S1, Figure S6).

## 4. Discussion

The increasing recognition of EVs as active players in physiological and pathological processes [11,28,29] also makes them attractive candidates for monitoring disease activity in multiple sclerosis. A number of studies utilized standard flow cytometry to demonstrate different levels of EVs present in the blood plasma of MS patients in the last two decades. Several studies report increased levels of endothelial EVs in patients in relapse [17,20,21,23,30] and other studies report increased levels of platelet and leukocyte EVs in different forms of MS compared to controls [22]. The goal of our study was to verify that RRMS patients attending hospital due to the current exacerbation of their symptoms can be distinguished from HC based on the count of cell-type specific plasma EVs. For the detection of endothelial EVs, we selected endoglin (CD105) as a marker which was successfully utilized for the demonstration of increased plasma endothelial EVs in paroxysmal nocturnal haemoglobinuria [31]. Endoglin is also expressed on macrophages [32], but in contrast to the widely used PECAM-1 (CD31) marker, it is not expressed by platelets [33]. As a red blood cell marker, we utilized the well-established glycophorin A (CD235a) [31,34]. The idea behind counting red blood cell EVs was to have a control population of EVs whose count would not be affected by MS disease activity. For the detection of platelet and lymphocyte populations, we double labeled EVs to increase its specificity. Platelet markers were thrombospondin receptor CD36 which besides their high expression on platelets, are also expressed on monocytes [35] and endothelial cells [36], and integrin subunit alpha IIb (CD41) which is expressed predominantly by platelets, but recently was also found on hematopoietic progenitors [37]. As a pan-leukocyte EVs marker, we utilized Protein Tyrosine Phosphatase Receptor Type C (CD45), which is expressed on the surface of all subsets of white blood cells [38] and is widely used in EVs analysis. The subpopulation of T-lymphocyte EVs was detected by marker T-cell receptor (CD3) and the subpopulation of B-lymphocyte EVs was detected using marker CD19 molecule [39]. Phosphatidylserine-positive EVs were detected using the well-established marker Annexin V [40,41].

To remove the signal of contaminating plasma lipoprotein particles [42,43,44], we isolated EVs prior to analysis by differential centrifugation. We assumed that the sedimentation would lead to the preferential retrieval of large EVs fitting in the sensitivity limit of our flow cytometer. The purity of isolated EVs was confirmed by cryo-TEM. Clusters of aggregated EVs or protein aggregates were not detected. The size of isolated EVs in an almost-native state varied between 70 and 500 nm with the majority of EVs being smaller than 300 nm. Infrequently, larger elongated EVs and spherical objects resembling EVs, but lacking sufficient structural details due to ice thickness, were identified. Our observation is compatible with a recent study demonstrating that only about 1% of EVs in plasma are larger than 300 nm [45]. To prevent the introduction of possible artifacts caused by the freezing of samples [46,47,48], we isolated and analyzed lEVs immediately after the collection of blood. In addition, fresh MS and HC samples were processed simultaneously on each measurement day. This labour-intensive approach was used to limit possible distortions of the results by unforeseen differences in processing MS and HC samples.

To increase the credibility of our flow cytometry analysis, we focused on particles which scattered enough light so as to be detected above the sensitivity limit of our cytometer. The lEVs gate was set up using silica beads with a refractive index comparable to the RI of EVs. The importance of utilizing beads with an RI similar to that of EVs for correct estimation of EVs size is demonstrated by the larger light scattering of 500 nm latex beads (RI = 1.59) than of 880 nm silica beads (RI = 1.43). As we did not measure the RI of plasma lEVs and some studies reported that the actual RI of EVs is smaller than silica beads [49], it is possible that lEVs detected in the gate were in fact larger than estimated by the ApogeeMix beads. Using these beads, we retrospectively determined that the approximate size of lEVs detected in the gate ranged from 650 to 2000 nm. The size of the detected lEVs probably prevented their visualization by cryo-TEM. All buffers used in EVs isolation were 100 nm filtered and produced minimal signals inside the lEVs gate. To exclude false positive and false double-positive events we included antibody wash [50]. The analysis of serial dilutions of labelled lEVs confirmed the linearity of lEVs counting, suggesting that the measurement is not notably affected by coincident (“swarm”) EVs detection [27]. However, the fluorescence intensity of the detected lEVs had a tendency to decrease with the dilutions, which is suggestive of coincident detection presence. Some level of coincident detection is unavoidable in EVs analyses with conventional flow cytometers [51]. Nevertheless, as there was no significant difference in the total detected events between the patient and control samples, we assume that coincident detection affected both experimental groups in a similar way. The performance of our method was validated by a parallel analysis of the plasma technical replicates. While double-labelled (CD36+CD41+) technical replicates provided satisfactory results, we noted that on one occasion, the discrepancy among the replicates labelled with Annexin V was higher than anticipated, reaching almost 30% of the replicates’ mean value. This suggests that despite the standardization of the sample handling, an occasional variation in the results may occur. While we believe that on a cohort level such occasional variation should not distort the results significantly, the values of individual samples must be interpreted carefully.

The number of all events detected in the lEVs gate was similar but varied more in MS patients than in HC. While it may be tempting to assign this higher variability to pathological conditions, it may also stem from different levels of blood lipoproteins. Lipoprotein particles such as HDL, LDL, VLDL and chylomicrons were shown to affect the results of EVs measurements by flow cytometry [42]. As healthy donors, but not patients, had been recommended a low-fat diet before blood collection, it is possible that the number of lipid particles contaminating isolated lEVs was higher in MS patients than in HCs [52]. In contrast to previously published reports, we did not find any difference in the number of fluorescently labelled lEVs. Counts of endothelial (CD105+), red blood cell (CD235a+), platelet (CD36+, CD41+), leukocyte (CD45+), T-lymphocyte (CD45+, CD3+), B-lymphocyte (CD45+, CD19+) and Annexin V+ lEVs were very similar in both MS patients and HC. Relative counts of endothelial and leukocyte lEVs were significantly lower in MS patients, but this difference must be interpreted with caution as we do not know if the count of all particles in the lEVs gate in MS patients and HC is affected by their different fasting status. In addition, the difference was in opposite direction to that anticipated in the previous reports [17,23,30]. The cohort of RRMS patients was heterogeneous in terms of therapeutic conditions, but uniform in one important clinical parameter—the recent exacerbation of symptoms. The majority of patients included in our study were taking disease modifying drugs, so it is possible that the effect of medication affected the number of lEVs produced during the disease relapse. While counts of lEVs in two nontreated patients included in our study did not deviate from the rest, lower counts of plasma EVs during the treatment of MS patients were reported previously [17,20,21,22,23,30]. The mean number of cell-specific lEVs were low, starting at ~23/µL for double positive CD45+CD19+ B-lymphocyte lEVs and ending at ~250/µL for most numerous CD235a+ red blood cell lEVs. Low numbers of detected lEVs contrast with normal counts of blood cells ~5 × 10^3^ leukocytes/µL, ~2 × 10^5^ platelets/µL and ~5 × 10^6^ red blood cells/µL. A recent study using a quantitative nanoparticle-tracking analysis estimated the presence of ~2 × 10^7^ EVs/µL of blood plasma with ~95% of them being smaller than 200 nm. The count of large EVs in the range 300–1000 nm was reported two orders of magnitude lower ~2 × 10^5^ EVs/µL [53], which is still 100 times higher than the mean number of all particles detected in the lEVs gate in our study (~1.6 × 10^3^ EVs/µL). The sum of the mean numbers of CD105+, CD235a+, CD41+ and CD45+ lEVs is about 600/µL in MS and 620/µL in HC, which represent just 37% and 55% of the detected events in the lEVs gate, respectively. It suggests that sizable portions of particles in the lEVs gate do not carry enough cell-specific markers to be detected by the antibodies utilized in our study.

To better understand the impact of lEVs isolation on the results of the analysis, we carried out a follow up study with frozen plasma supernatant depleted of lEVs. Interestingly, in lEV-depleted plasma, we detected almost ~6 × 10^4^ events per µL in the lEVs gate, which is about 50 times more than in the isolated fraction of fresh lEVs. So, either the majority of plasma particles able to provide a signal in the lEVs gate did not sediment during the isolation of lEVs or the subsequent freezing of plasma supernatant introduced artificial signals. Cryo-TEM of lEV-depleted plasma revealed the presence of electron-dense particles with a size of up to 300 nm lacking a phospholipid bilayer, likely lipoproteins [54]. The particles were also observed in isolated lEVs, but infrequently. These electron-dense, yet light particles could explain the events detected in the lEVs gate especially if their RI is higher than the RI of EVs. A method capable of distinguishing lipoproteins from EVs based on their higher RI was recently reported [49]. In addition, the lEV-depleted plasma contained small particles with a size up to 50 nm and protein aggregates which were not observed in isolated lEVs. No wash method was used for the labelling of plasma depleted of lEVs and we experienced high, nonspecific signals produced by all IgG2a antibodies. In addition, the discrimination of fluorescence-negative and positive populations had to be performed subjectively with every sample as the threshold produced by isotypic controls did not fit the distribution of collected events. Surprisingly, the counts of fluorescently labelled events in the lEVs gate were quite similar as in isolated lEVs and again the sample of MS patients did not differ from HC. The only exception was modest (~48%) elevation of Annexin V-positive events, suggesting an increased formation of phosphatidylserine-positive EVs in relapsing RRMS patients. The reason for this observation is not clear as no difference in the number of Annexin V-positive events in the isolated lEVs was identified. In addition, the overlap of values with HC was substantial, thereby precluding the decisive identification of individual samples and prompting the verification of this finding in further studies.

## 5. Conclusions

Our data do not support the hypothesis that the exacerbation of the disease in RRMS patients leads to increased numbers of circulating plasma lEVs which can be monitored by standard flow cytometry. The presence and purity of isolated EVs in our study was confirmed by cryo-TEM. However, the low sensitivity of standard flow cytometry limited their analysis to a small segment of the largest EVs, with a size starting at 650 nm, which scatter more light than the 590 nm silica beads, and may not reflect MS pathophysiology. In such cases, the diagnostic potential of the standard flow cytometry analysis of lEVs in plasma might be limited. Recently, a similar conclusion was reached for the quantification of large phosphatidylserine-positive EVs in CSF of MS patients [55]. The results of our study differ from previously published reports and support the ongoing effort for the technological improvement of cytometry sensitivity and the standardization of EV-analysis protocols [56,57].

## Figures and Tables

**Table 1:** Analysis of fresh lEVs isolated from blood plasma of MS patients and healthy controls. Results of flow cytometry analysis of lEVs isolated from blood plasma of MS patients and healthy controls by differential centrifugation. “All events” and “events in lEVs gate” represent event counts recorded “within and outside” or “only within” the lEVs gate, respectively. Left side of the table represent counts of labelled lEVs detected in µL of plasma. Right side of table represents relative numbers of lEVs related to all events detected in lEVs gate (%). Mean values with 95% confidence interval are presented. MS patients (*n* = 15); healthy controls (*n* = 16 or * *n* = 15).

Analysis of Fresh lEVs Isolated from Blood Plasma of MS Patients and Healthy Controls										
	Count of Positive lEVs/µL of Plasma					% of Positive lEVs				
Labelling	Patient	95% CI	Control	95% CI	*p* < 0.05	Patient	95% CI	Control	95% CI	*p* < 0.05
All events	9783	4884–14,683	7659	5554–9764	no	---		---		no
Events in lEVs gate	1620	576–2663	1116	789–1443	no	15.1	12.9–17.4	15.0	12.6–17.4	no
Annexin V	212	115–308	203	132–275	no	17.0	12.4–21.5	20.9	17.5–24.2	no
CD105	54	34–74	72	41–103	no	4.5	3.4–5.5	7.6	5.7–9.5	yes
CD235a	250	178–323	244	160–328	no	24.6	19.2–30.1	26.4	19.8–33.1	no
CD36	226	178–274	236	168–304	no	19.3	14.2–24.4	25.1	19.0–31.3	no
CD41	171	132–211	180	124–235	no	14.3	10.3–18.2	18.6	14.7–22.5	no
CD36 + CD41	131	101–160	146	98–193	no	11.4	7.8–14.9	15.4	11.6–19.1	no
CD45 *	108	75–140	126	93–159	no	8.4	6.4–10.4	12.3	9.1–15.4	yes
CD19 *	50	27–74	81	36–126	no	3.4	2.6–4.2	6.7	4.1–9.3	yes
CD3 *	95	57–133	166	87–245	no	7.1	4.9–9.3	14.3	9.4–19.3	yes
CD45 + CD19 *	23	13–34	45	16–74	no	1.9	1.1–2.7	3.9	2.0–5.7	no
CD45 + CD3 *	49	28–70	79	46–112	no	4.1	2.2–6.0	7.4	5.1–9.6	yes

## Data Availability

The data used to support the findings of the presented study are available from the corresponding author upon request.

## Supplementary Materials

The following supporting information can be downloaded at: https://www.mdpi.com/article/10.3390/jcm11102832/s1, Supplementary Text S1: FACS flow rate measurement, Text S2: Preparation of compensation samples, Text S3: Labelling and analysis of lEVs Deprived Plasma, Text S4: Results of analysis of plasma deprived of lEVs, Table S1: Analysis of blood plasma depleted of lEVs by centrifugation, Figure S1: Validation of flow cytometry analysis of isolated lEVs. Figure S2: Comparison of ApogeeMix beads in scattergram with arbitrary units of our cytometer and in scattergram with calibrated standard units, Figure S3: Counts of isolated fresh lEVs in blood plasma of MS patients and HC, Figure S4: Relative numbers of labelled isolated fresh lEVs in MS patients and HC (data not present in Figure 5), Figure S5: Illustrative scattergrams of PBS-BSA buffer and Annexin V binding buffer used for lEVs dilution, Figure S6: Analysis of frozen plasma depleted of lEVs by centrifugation.
