# Supplementary material for: Flow Cytometry Analysis of Blood Large Extracellular Vesicles in Patients with Multiple Sclerosis Experiencing Relapse of the Disease
Source: J Clin Med. 2022 May 17;11(10):2832. doi: 10.3390/jcm11102832 (PMC9145450; doi:10.3390/jcm11102832)
Supplement: Supplementary file 1 [file jcm-11-02832-s001.zip › jcm-1660412-supplementary.pdf]

Text S1: FACS flow rate measurement

Flow rate was measured by Fluoresbrite® YG Microspheres, Calibration Grade 6.00 µm, cat. No. 18862-1, Polysciences diluted in 0.1% BSA in PBS. Bead count in solution was calculated under microscope using Bürker chamber slide. Two distinct dilutions were prepared in triplicates and measured on cytometer. Mean flow rate was 9.2 µl/min with standard deviation 0.37 µl/min.

Text S2: Preparation of compensation samples

Aliquots of EDTA anticoagulated blood (40 µl) were mixed with 10 µl of antibody solutions in following manner: CD4 PE; CD3 FITC; CD19 APC; CD4 PE + CD19 APC or PBS-BSA. Samples were incubated in dark at RT for 20 minutes and lysed with 10 volumes of BD FACS Lysing solution for 10 minutes. Lysed blood was diluted with 1 ml of PBS-BSA and leukocyte pelleted by centrifugation at 500 g, 22°C for 10 minutes. The cells were resuspended in PBS-BSA solution and analysed on cytometer.

Text S3: Labelling and analysis of lEVs Deprived Plasma

Frozen samples of lEV deprived plasma were thawed on ice and vortexed. Samples were labelled in a similar manner as isolated lEVs, but without washing (Figure 2). Forty-five µl of lEV deprived plasma were incubated with 15 µl of mAb solutions in dark at RT for 20 min. Annexin V labelling was carried in plasma supplemented with ABB (1x final concentration). The samples were diluted with 1 ml of PBS-BSA or ABB and directly analysed using flow cytometry. In lEVs depleted plasma samples fluorescence threshold was set on each sample (between negative and positive events) since washing was omitted.

Text S4: Results of analysis of plasma deprived of lEVs

We analysed the presence of particles in frozen plasma supernatants after sedimentation of lEVs (Figure 2). Samples were labelled by no wash protocol and we used identical gating strategy to create lEVs gate (Figure S6A). Although the plasma was depleted of large vesicles (14 000 g, 70 minutes) a surprisingly high number of events was recorded in lEVs gate (59 490 ± 34 540 particles/µl in MS patients and 57 931 ± 53 257 particles/µl in HC). Number of Annexin V positive particles in lEVs gate was significantly higher in MS patients than in HC: 241 ± 62 particles/µl vs 161 ± 35 particles/µl ($p = 0.0312$, Figure S6B and C). No other significant differences were detected (Table S1). We also noticed a strong nonspecific binding of IgG2a antibodies (CD105 and CD3) to particles present in lEVs depleted plasma which was not present in samples of isolated lEVs (not shown). As elevated binding was also recorded for IgG2a isotypic control antibody we did not include the data into the analysis.

Table S1

Analysis of frozen plasma depleted of lEVs of MS patients and healthy controls

	count of positive particles/ µl of plasma					% of Positive Particles				
Positivity	Patient	95% CI	Control	95 % CI	P < 0.05	Patient	95% CI	Control	95 % CI	P < 0.05
All events	836 615	481 120 – 1 192 109	552 547	158 281 – 1 042 909	no	---		---		no
Events in lEVs gate	59 490	24 950 – 94 029	57 931	4665 - 111197	no	6.2	4.8 - 7.7	8.7	7.3 - 10.2	yes
Annexin V	241	179 - 304	161	122 - 200	yes	0.5	0.3 - 0.6	0.4	0.3 - 0.6	no
CD105	X		X		no	X		X		no
CD235a	267	152 - 382	186	132 - 240	no	0.6	0.4 - 0.9	0.9	0.5 - 1.3	no
CD36	190	88 - 292	116	86 -147	no	0.4	0.3 - 0.6	0.5	0.3 - 0.8	no
CD41	197	111 - 284	147	94 - 198	no	0.5	0.3 - 0.6	0.6	0.3 - 0.9	no
CD36 + CD41	43	21 - 64	29	18 - 47	no	0.1	0.1 - 0.2	0.2	0.1 - 0.3	no
CD45	190	52 - 329	202	101 - 302	no	0.4	0.3 - 0.6	0.9	0.4 - 1.5	no
CD19	211	138 - 284	385	-11 - 781	no	0.6	0.4 - 0.8	1.1	0.5 - 1.7	no
CD3	X		X		no	X		X		no
CD45 + CD19	22	16 - 28	42	5 - 79	no	0.1	0.0 - 0.1	0.2	0.1 - 0.3	no
CD45 + CD3	X		X		no	X		X		no

Table S1: Analysis of blood plasma depleted of lEVs by centrifugation. Results of flow cytometry analysis of blood plasma of MS patients and healthy controls depleted of lEVs by differential centrifugation. "All events" and "events in lEVs gate" represent event counts recorded "within and outside" or "only within" the lEVs gate, respectively. Left side of the table represent counts of labelled particles detected in µl of plasma. Right side of table represent relative numbers of labelled particles related to all events detected in lEVs gate (%). Mean values with 95% confidence interval are presented. MS patients (n = 15); healthy controls (n = 16). X = results omitted due to presence of non-specific signal of IgG2a antibodies.

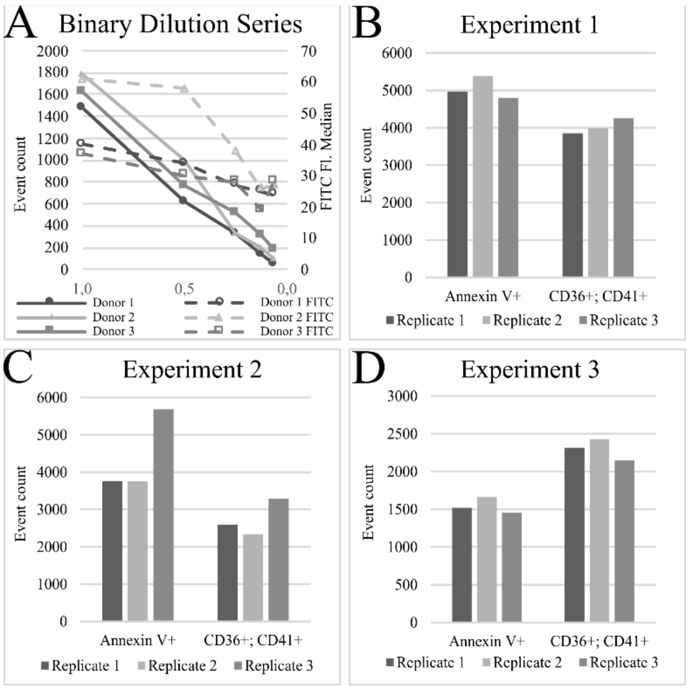

Figure S1. Validation of flow cytometry analysis of isolated lEVs. A. Binary dilution series of isolated plasma vesicles of 3 different donors. Counted events are double positive for CD36 and CD41 markers (full line). Dashed lines are CD 36 fluorescence medians in dilutions. B-D. Three separate experiments of plasma lEVs analysis performed in three technical replicates of isolation and labelling using Annexin V or CD36 + CD41. Counts represent labelled events recorded in lEVs gate within 2 min of analysis.

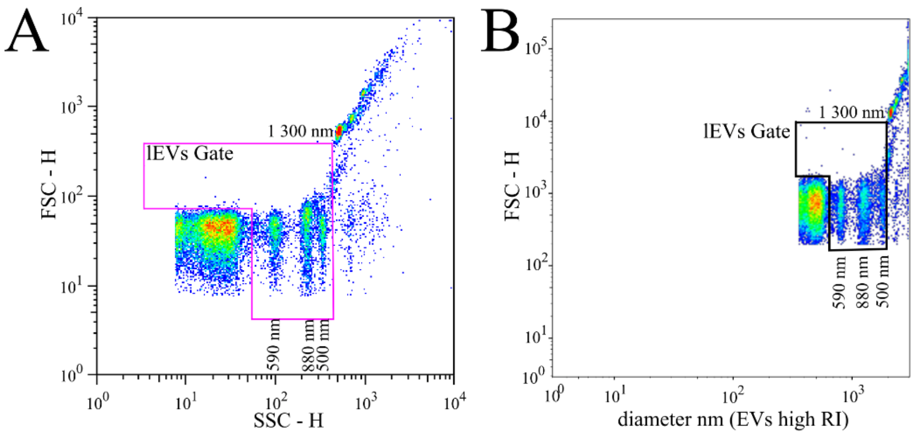

Figure S2. Comparison of ApogeeMix beads in scattergram with arbitrary units of our cytometer (A) and in scattergram with calibrated standard units (B). Size of each beads population and placement of lEVs gate are shown in scattergrams.

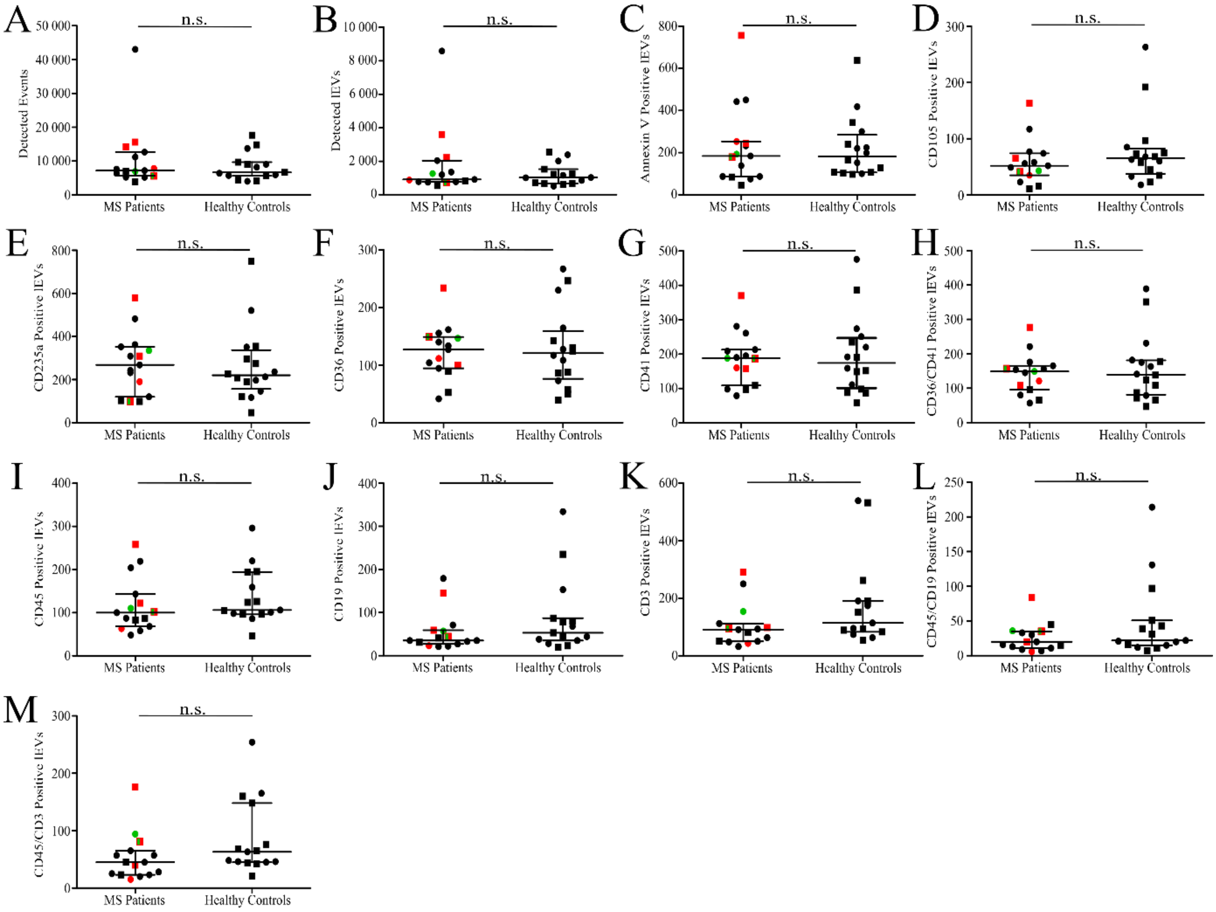

Figure S3. Counts of isolated fresh lEVs in blood plasma of MS patients and HC. Number of all events (A) and number of events inside lEVs gate (B) collected during the analysis. Counts of Annexin V+ (C), endothelial CD105+ (D), erythrocyte CD235a+ (E), platelet CD36+ (F), platelet CD41+ (G), platelet CD36+CD41+ (H), leukocyte CD45+ (I) $^{\#}$, B-lymphocyte CD19+ (J) $^{\#}$ and CD45+CD19+ (L) $^{\#}$, T-lymphocyte CD3+ (K) $^{\#}$ and CD45+CD3+ (M) $^{\#}$ lEVs in µl of plasma. The line represents median of values with interquartile range. Women (circles), men (squares), patients without treatment (green), patient which received intravenous corticoids up to 14 days before blood collection (red). MS patients (n = 15) and HC (n = 16 or $^{\#}$n = 15). Non-significant (n.s.).

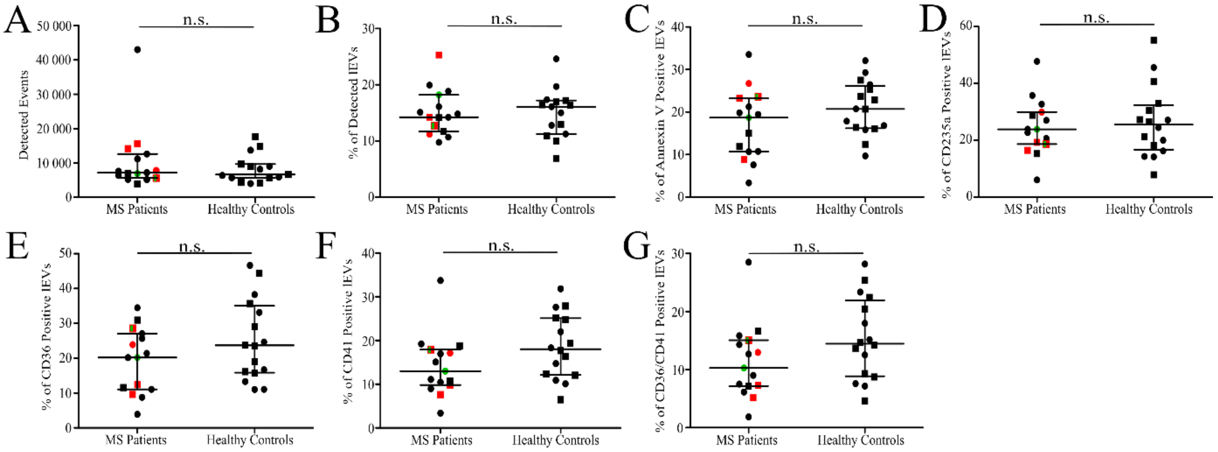

Figure S4. Relative numbers of labelled isolated fresh lEVs in MS patients and HC (data not present in Fig. 5). Number of events collected in lEVs gate (A), percentage of events in lEVs gate out of all collected events (B). Relative numbers of Annexin V+ (C), erythrocyte CD235a+ (D), platelet CD36+ (E), platelet CD41+ (F) and platelet CD36+CD41+ (G) lEVs out of all events collected in lEVs gate. The line represents median of values with interquartile range. Women (circles), men (squares), patients without treatment (green), patient which received intravenous corticoids up to 14 days before blood collection (red). MS patients (n = 15) and HC (n = 16). Non-significant (n.s.).

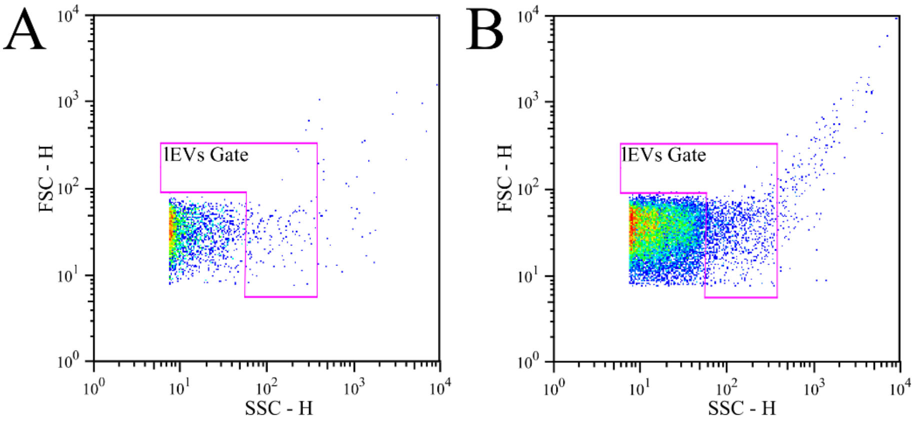

Figure S5. Illustrative scattergrams of PBS-BSA buffer (A) and Annexin V binding buffer (B) used for lEVs dilution. lEVs gate is placed in both scattergrams.

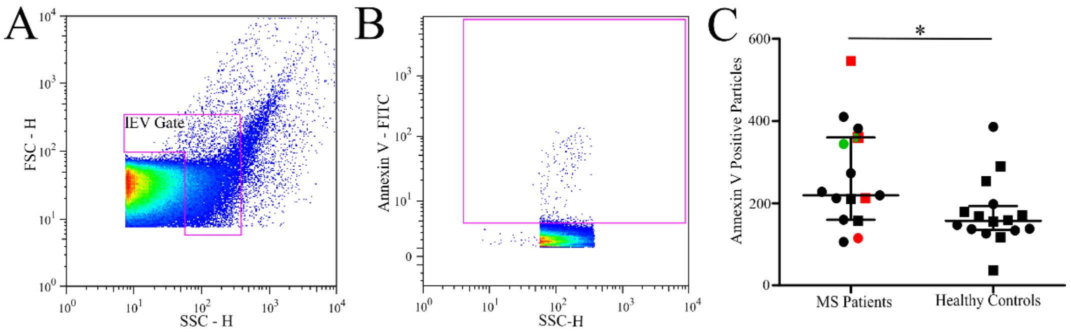

Figure S6. Analysis of frozen plasma depleted of lEVs by centrifugation. A. An illustrative scattergram of particles present in plasma deprived of lEVs. B. Representative fluorescence dotplot of Annexin V labelled particles in lEVs gate. C. Number of Annexin V+ particles in lEVs gate in μl of plasma deprived of lEVs. The line shows median of values with interquartile range. Women (circles), men (squares), patients without treatment (green), patient which received intravenous corticoids up to 14 days before blood collection (red). MS patients (n = 15), HC (n = 16), *$p < 0.05$.
